# Supplementary material for: Patterns of Intron Gain and Loss in Fungi
Source: PLoS Biol. 2004 Nov 30;2(12):e422. doi: 10.1371/journal.pbio.0020422 (PMC532390; doi:10.1371/journal.pbio.0020422)
Supplement: Table S1 — Also available at http://genes.mit.edu/NielsenEtAl/. (4.3 MB ZIP). [file pbio.0020422.st001.zip › NielsenEtAl/html/1418.html]

AN8275.1.NCU01692.1.MG07202.1.FG01422.1


```
 CLUSTAL W (1.82) Multiple Sequence Alignments - Introns Inserted


Sequence 1: NCU01692.1	469 aa
Sequence 2: MG07202.1	473 aa
Sequence 3: FG01422.1	470 aa
Sequence 4: AN8275.1	474 aa
Alignment Length: 475 aa
Number Identitical Residues: 355 aa
Alignment Score (without introns) 15052


MG07202.1 	MASAMRLSSAFRATARAPAFTARR-TAFNGMRCYSA-KTQ0TLKERFAELLPEKIEEIKS
NCU01692.1	MAPVMRLGSAALRSS--IHLTSRQ-TAFTAARCYSS-KTQ0TLKERFAELLPENIEKIKA
FG01422.1 	MASVARLSNAALRAS-LRSSPVNG-SAFNAIRCYSA-KTQ0TLKERFAELLPEKIEQVKA
AN8275.1  	MASTLRLSTSALRSSTLAGKPVVQSVAFNGLRCYSTGKTK0SLKETFADKLPGELEKVKK
          	**.. **..:   ::     .   : **.. ****:.**: :*** **: ** ::*::* 

MG07202.1 	LRK2EHGSKVIDKVTLDQVYGGARGIKCLVWE~GSVLDSEEGIRFRGKT~IPECQELLPK
NCU01692.1	LRK2EHGSKVVDKVTLDQVYGGARGIKCLVWE~GSVLDAEEGIRFRGKT~IPECQELLPK
FG01422.1 	LRK2EHGSKVVDKVTLDQVYGGARGIKALVWE~GSVLDSEEGIRFRGKT~IPECQELLPK
AN8275.1  	LRK2EHGNKVIGELTLDQAYGGARGVKCLVWE0GSVLDSEEGIRFRGLT0IPECQKLLPK
          	*** ***.**:.::****.******:*.**** *****:******** * *****:****

MG07202.1 	APGGKEPLPEG1LFWLLLTGEVPSEAQVRALSAEWAARSDLPKFVEELIDRCPNDLHPMA
NCU01692.1	APGGKEPLPEG1LFWLLLTGEVPSEQQVRDLSAEWAARSDVPKFIEELIDRCPSDLHPMA
FG01422.1 	APGGKEPLPEG1LFWLLLTGEVPTEQQVRDLSAEWAARSDLPKFVEELIDHCPTDLHPMA
AN8275.1  	APGGEEPLPEG1LFWLLLTGEVPSEQQVRDLSAEWAARSDLPKFIEELIDRCPSTLHPMA
          	****:****** ***********:* *** **********:***:*****:**. *****

MG07202.1 	QFSMAITALEQTSSFAKAYAKGINKKEYWGYTFEDSMDLIAKLPTIAARIYQNVFKGGKV
NCU01692.1	QLSLAVTALEHTSSFARAYAKGINKKEYWGYTFEDSMDLIAKLPTIAARIYQNVFKGGKV
FG01422.1 	QFSLAVTALEHTSSFAKAYAKGINKKEYWGYTFEDSMDLIAKLPNIASRIYQNVFKGGKV
AN8275.1  	QFSLAVTALEHESAFAKAYAKGINKKEYWHYTFEDSMDLIAKLPTIAAKIYRNVFKDGKV
          	*:*:*:****: *:**:************ **************.**::**:****.***

MG07202.1 	APVQKDKDYSFNFANQLGFGNNSDFVELLRLYLTIHTDHEGGNVSAHTTHLVGSALSSPF
NCU01692.1	AAVQKDKDYSFNFANQLGFGDNKDFVELLRLYLTIHTDHEGGNVSAHTTHLVGSALSSPF
FG01422.1 	AAIQKDKDYSFNFANQLGFADNADFVELMRLYLTIHTDHEGGNVSAHTTHLVGSALSSPF
AN8275.1  	APIQKDKDYSYNLANQLGFADNKDFVELMRLYLTIHSDHEGGNVSAHTTHLVGSALSSPM
          	*.:*******:*:******.:* *****:*******:**********************:

MG07202.1 	LSLAAGMNGLAGPLHGL~ANQEVLNWLTEMKKSVGDDLSDKSITDYLWSTLNSGRVVPGY
NCU01692.1	LSVAAGLNGLAGPLHGL~ANQEVLNWLTEMKKVIGDDLSDEAITKYLWDTLNAGRVVPGY
FG01422.1 	LSLAAGLNGLAGPLHGL~ANQEVLNWLTEFKKSVGDDLSDKAITDYLWSTLNAGRVVPGY
AN8275.1  	LSLAAGLNGLAGPLHGL2ANQEVLNWLTEMKKVVGNDLSDQSIKDYLWSTLNAGRVVPGY
          	**:***:********** ***********:** :*:****::*..***.***:*******

MG07202.1 	GHAVLRKTDPRYTAQRVFAQE1KMPNDPMFKLVSQVYKIAPGVLTEHGKTKNPFPNVDA~
NCU01692.1	GHAVLRKTDPRYSAQRKFAQE~HLPEDPMFQLVSQVYKIAPKVLTEHGKTKNPYPNVDA~
FG01422.1 	GHAVLRKTDPRYMAQRTFAQE~KMPNDPMFKLVSQVYKIAPGVLTEHGKTKNPYPNVDA~
AN8275.1  	GHAVLRKTDPRYTSQREFALR~KLPDDPMFKLVSQVYKIAPGVLTEHGKTKNPYPNVDA0
          	************ :** ** . ::*:****:********** ***********:***** 

MG07202.1 	HSGVLLQYYGLTEANYYTVLFGVSRAIGVLPQLIIDRAVGAPIER2PKSFSTEKWIEIAK
NCU01692.1	HSGVLLQHYGLTEANYYTVLFGVSRAIGVLPQLIIDRAVGAPIER2PKSYSTDKWIEICK
FG01422.1 	HSGVLLQYYGLTEANYYTVLFGVSRAIGVLPQLIIDRALGAPIER~PKSFSTDKWAELVK
AN8275.1  	HSGVLLQYYGLTEANYYTVLFGVSRALGVLPQLIIDRAFGAPIER~PKSFSTEAYAKLVG
          	*******:******************:***********.****** ***:**: : ::  

MG07202.1 	KTAA
NCU01692.1	KL--
FG01422.1 	KL--
AN8275.1  	AKL-
          	
```
